# Two-Year Event-Free Survival Prediction in DLBCL Patients Based on *In Vivo* Radiomics and Clinical Parameters

**DOI:** 10.3389/fonc.2022.820136

**Published:** 2022-06-08

**Authors:** Zsombor Ritter, László Papp, Katalin Zámbó, Zoltán Tóth, Dániel Dezső, Dániel Sándor Veres, Domokos Máthé, Ferenc Budán, Éva Karádi, Anett Balikó, László Pajor, Árpád Szomor, Erzsébet Schmidt, Hussain Alizadeh

**Affiliations:** ^1^Department of Medical Imaging, Medical School, University of Pécs, Pécs, Hungary; ^2^Medical University of Vienna, Center for Medical Physics and Biomedical Engineering, Vienna, Austria; ^3^University of Kaposvár, PET Medicopus Nonprofit Ltd., Kaposvár, Hungary; ^4^Department of Biophysics and Radiation Biology, Faculty of Medicine, Semmelweis University, Budapest, Hungary; ^5^In Vivo Imaging Advanced Core Facility, Hungarian Centre of Excellence for Molecular Medicine, Budapest, Hungary; ^6^Institute of Transdisciplinary Discoveries, Medical School, University of Pécs, Pécs, Hungary; ^7^Institute of Physiology, Medical School, University of Pécs, Pécs, Hungary; ^8^Department of Hematology, University of Kaposvár, Kaposvár, Hungary; ^9^County Hospital Tolna, János Balassa Hospital, Szekszárd, Hungary; ^10^Department of Pathology, Medical School, University of Pécs, Pécs, Hungary; ^11^1st Department of Internal Medicine, Medical School, University of Pécs, Pécs, Hungary

**Keywords:** DLBCL, radiomics, [^18^F]FDG PET/CT, automated machine learning, tumor imaging

## Abstract

**Purpose:**

For the identification of high-risk patients in diffuse large B-cell lymphoma (DLBCL), we investigated the prognostic significance of *in vivo* radiomics derived from baseline [^18^F]FDG PET/CT and clinical parameters.

**Methods:**

Pre-treatment [^18^F]FDG PET/CT scans of 85 patients diagnosed with DLBCL were assessed. The scans were carried out in two clinical centers. Two-year event-free survival (EFS) was defined. After delineation of lymphoma lesions, conventional PET parameters and *in vivo* radiomics were extracted. For 2-year EFS prognosis assessment, the Center 1 dataset was utilized as the training set and underwent automated machine learning analysis. The dataset of Center 2 was utilized as an independent test set to validate the established predictive model built by the dataset of Center 1.

**Results:**

The automated machine learning analysis of the Center 1 dataset revealed that the most important features for building 2-year EFS are as follows: max diameter, neighbor gray tone difference matrix (NGTDM) busyness, total lesion glycolysis, total metabolic tumor volume, and NGTDM coarseness. The predictive model built on the Center 1 dataset yielded 79% sensitivity, 83% specificity, 69% positive predictive value, 89% negative predictive value, and 0.85 AUC by evaluating the Center 2 dataset.

**Conclusion:**

Based on our dual-center retrospective analysis, predicting 2-year EFS built on imaging features is feasible by utilizing high-performance automated machine learning.

## Introduction

Non-Hodgkin lymphoma (NHL) is globally the most common hematological malignancy, accounting for nearly 3% of cancer diagnoses and deaths (1). The NHLs are a diverse group of malignancies, about 80% of which are of B-cell origin (B-NHL) in the Western hemisphere. The most common histologic subtype in adults worldwide is diffuse large B-cell lymphoma (DLBCL), comprising about 30%–40% of NHLs diagnosed each year (2, 3). DLBCL comprises a heterogeneous group of diseases with different biology, clinical presentations, and response to treatment (4, 5). DLBCL is potentially curable with standard treatment in 50%–60% of cases. About 25%–30% of patients are resistant to standard chemo-immunotherapy; therefore, other therapeutic approaches are utilized, namely, 20% of patients are treated with salvage therapy including high-dose therapy and autologous hematopoietic stem cell transplantation (4, 6, 7).

Advances on the understanding of the genetic landscape and molecular features of DLBCL have identified high-risk groups with poor response to chemo-immunotherapy. There is an unmet clinical need to identify these high-risk patients as early as possible in order to apply targeted and more intensive therapy on individualized basis, as the majority of refractory or relapsed patients will eventually die from their disease. The initial evaluation of DLBCL patients is aimed at determining the stage of the disease and assessing for end-organ damage either by the disease and/or preexisting comorbid conditions. The workup in a patient with suspected lymphoma usually starts with comprehensive chemistry panel including complete blood counts with differentials, hemostasis parameters, renal function, hepatic function, lactate dehydrogenase enzyme (LDH), beta-2-microglobulin (B2M), hepatitis B and C, Epstein–Barr Virus (EBV), and human immunodeficiency viral serology. Lymph node biopsy is required to establish a definitive diagnosis of lymphoma; this should be an excisional biopsy rather than a needle biopsy, because nodal architecture is often difficult to assess when small amounts of tissue are used (8–10).

After the histologic confirmation of DLBCL, imaging study is requested to assess the extent and stage of disease. The preferred imaging modality is functional imaging with 2-deoxy-2-[^18^F]fluoro-D-glucose [(^18^F)(FDG)] positron emission tomography/x-ray computed tomography (PET/CT) (hereinafter referred to as [^18^F]FDG PET/CT). This modality is the mostly used at baseline, prior to the start of treatment and for monitoring the efficacy of therapy (11–13).

Taking into consideration that about 20%–25% of patients are primarily resistant to the current 1st-line treatment with rituximab-based chemo-immunotherapy (14), identifying the high-risk group that does not respond has very high priority. One of these modalities could be the use of conventional and textural parameters derived from the baseline [^18^F]FDG PET/CT. Methods to individualize treatment choices are being increasingly employed in different clinical trials, yielding favorable correlations with improved response rates (5, 15). Studies in the field of cancer imaging research have been actively engaged with radiomics in combination with machine learning (16). However, radiomics has been reported to be sensitive to various factors such as individual biology, acquisition protocols, choice of delineation, binning and resolution, as well as calculation methods, which challenge prior studies to repeat (17). Nevertheless, standardization proposals such as the Imaging Biomarker Standardization Initiative (IBSI) (18) support the endeavor to report findings in a repeatable way.

In DLBCL patients, disease characteristics and outcomes vary widely, pointing to the importance of patient’s classification through identification of sensitive prognostic features especially prior to the start of therapy. For this purpose, we have tried to elucidate the prognostic significance of metabolic heterogeneity (19). We have highlighted metabolically active tumor volume and standardized uptake value (SUV)-based parameters such as SUV-max, SUV-min, total metabolic tumor volume (TMTV), and total lesion glycolysis (TLG) and compared their applicability with other radiomic parameters as well as clinical and pathological data.

We hypothesize that 2-year event-free survival (EFS) prediction models built on these features are feasible by utilizing automated machine learning in a multi-center environment. Hence, the objectives of this study were (a) to collect a dual-center dataset including conventional PET, radiomics, and clinical parameters of DLBCL patients; (b) to build a 2-year EFS prediction model by using one center data; and (c) to validate the established model by an independent dataset coming from another center.

## Materials and Methods

### Patient’s Data

The baseline pretreatment [^18^F]FDG PET/CT scans of 85 patients diagnosed with DLBCL performed in the period between January 2014 and December 2019 were assessed. The [^18^F]FDG PET/CT scans were carried out in two centers: at University of Pécs, Department of Medical Imaging—Center 1 including 41 patients, and at University of Kaposvár, Hungary—Center 2 including 44 patients. The median age of patients in this study population was 59 years (range: 23–81 years) with 48.20% (*n* = 41) of patients older than 60. In this cohort, 40 (47%) patients were male, and 45 (53%) were female. The patients with incomplete medical records and those who received non-standard treatments were excluded from the final analysis. The Eastern Cooperative Oncology Group (ECOG) performance status >2 was reported in 27 (31.80%) cases (in 2 cases, the ECOG status was unknown) with ECOG status unknown in 2 patients. All patients were treated with standard R-CHOP-21 treatment regimen for at least 4 full cycles. The patients were classified to germinal center B-cell-like (GCB) or activated B-cell (non-GCB) type using the Hans algorithm (20). The data regarding the cell of origin (COO) (based on the Hans algorithm) were available in 82 patients; 29 (37.60%) were GCB and 53 (62.40%) were non-GCB. The clinical stage was evaluated by the modified Ann Arbor and Lugano classification.

The pathological and clinical data and the Revised International Prognostic Index (R-IPI) were also determined before the initiation of the therapy (R-IPI: 0: 8, 1: 15, 2: 23, 3: 27, 4: 12 patients).

EFS was defined as the time from registration date to disease relapse, progression, or death related to the lymphoma. Complete response (CR), partial response (PR), progression (PD), refractory disease, and relapse were defined according to the International Working Group response criteria for lymphoma (11, 21).

### [^18^F]FDG PET/CT Studies

Pretreatment whole-body [^18^F]FDG PET/CT scans were performed using a Mediso AnyScan 16 PET/CT scanner in 41 patients (Center 1) and a Siemens Biograph Truepoint 64 PET/CT scanner in 44 patients (Center 2). All patients in the study were subjected to full history and complete clinical examination including the clinical stage of the disease. The patients were instructed to fast for 6 h before the scan. Blood glucose level was ensured to be below <8 mmol/L in all patients before the injection of radiotracer. Intravenous (i.v.) injection of [^18^F]FDG through an i.v. line with a dose of 3–4 MBq/kg was administered. After tracer injection, the patient was asked to stay for at least 60 min in a dark room covered by warm blankets. No speaking, chewing, or reading was allowed.

During a PET-CT examination in Center 1, we execute a low-dose CT scan first with the following parameters: x-ray tube voltage: 120 kVp (depending on the patient’s size, 140 kVp is used in bariatric patients), x-ray tube current: 24–26 mAs (also depending on the patient’s size, a higher tube current can be applied in bariatric patients), pitch: 1.5, and slice thickness: 2.5 mm. In order to achieve attenuation correction and accurate body mapping, the CT series has to cover the whole PET range of patients from skull to mid-thighs. After this step, the PET acquisition follows the CT series without delay. We have applied the 3D acquisition method for PET data collection with a 3 min frame time. Usually, between 7 and 10 bed positions can cover a general scan range, by axial FOV: 15.12 cm (longitudinal FOV in the patient’s *z*-axis). According to the manufacturer’s recommendations, the PET images were iteratively reconstructed using the Tera-Tomo™ 3D image reconstruction algorithm in a 167 × 167 × 234 matrix, which resulted in an isotropic voxel size of 4 mm.

In the Center 2 PET-CT examination, we execute a low-dose CT scan first with the following parameters: x-ray tube voltage: 120 kVp (depending on the patient’s size, 140 kVp is used in bariatric patients), x-ray tube current: reference effective mAs: 60 using CareDose, pitch: 1.5, and slice thickness: 5 mm. In order to achieve attenuation correction and accurate body mapping, the CT series shall cover the whole PET range of patients from skull to mid-thigs. After this step, the PET acquisition follows the CT series without delay. We apply 3D mode acquisition for PET data collection with 3 min frame time. Usually, between 7 and 9 bed positions can cover a general scan range, by axial FOV: 16.2 cm (longitudinal FOV in the patient’s *z*-axis). PET images were iteratively reconstructed using the 2D OSEM (3i8s, 5 mm Gaussian filtering) image reconstruction algorithm in a 168 × 168 matrix.

### Delineation and Feature Extraction

Lymphoma lesions were detected by InterView FUSION ver. 3.10 (Mediso Medical Imaging Systems Ltd., Budapest, Hungary) clinical evaluation software. The average SUV-max value of the liver (3.5–5.5) served as a reference threshold for the semi-automated algorithm (22). This approach was selected to minimize the effects of patient-specific radiotracer distributions (23). The average of three randomly placed volumes of interest (VOIs) from the unaffected liver regions was used. After the execution of the algorithm with the selected parameters from the automatically segmented regions, the non-affected regions, such as regions with physiological activity (urine in kidneys or in the bladder, or brain activity) or radiotracer accumulations, which are not related to the lymphoma (such as bowel uptake caused by metformin intake), were manually excluded. TLG, TMTV, and SUV-max were automatically calculated across all delineated lesions. Furthermore, SUV-peak values were segmented from the VOI with the highest activity. For further radiomic feature extraction, the largest VOI was selected in each patient. From each of these VOIs, IBSI radiomic features including intensity, histogram, morphological, neighborhood gray-tone difference matrix (NGTDM), gray-level co-occurrence matrix (GLCM), gray-level run length matrix, (GLRLM), and gray-level size zone matrix (GLSZM) features were extracted. For the IBSI-conform reporting details of the radiomic analysis, see **Supplementary Table 1**.

### Reference Standard

During the follow up, 2-year EFS was chosen as a clinically relevant cutoff point (24). Based on this criterion, patients were selected into two groups. In Group 0, the patients had no events during the 2-year follow up, and in group 1, the patients had primary refractory disease or relapsed during the 2-year period.

### Statistical Analysis

A chi-square test was used for the assessment of binary variables *via* SPSS (SPSS statistical software 27). First, data from both centers were evaluated together based on 2-year EFS. A significant association was sought between the two groups, defined above and the following clinical data: sex, stage, R-IPI, and COO. Data were also separated by the two centers, where the [^18^F]FDG PET/CT scans were performed. A significant relationship was sought between the two centers and the two clinical outcomes, stages, R-IPI values, and COO. The test results were considered statistically significant if the *p*-value was under 0.05.

### Automated Machine Learning Analysis and Biomarker Identification

The Center 1 dataset was utilized as a training set, given that it had more balanced remission–progression subgroups compared to Center 2 (**Table 2**). The dataset underwent automated machine learning analysis from the Dedicaid AutoML Research package (Dedicaid GmbH, Vienna, Austria). This step included automated data preprocessing for redundancy reduction, class imbalance reduction, as well as feature engineering, ranking, and selection (25). The data were split into 100-fold *via* random subsampling (26), and mixed ensemble learning was applied in each fold to generate a model predicting the final 2-year EFS. For quality control, the AutoML approach also performed a single-center cross-validation across the 100-fold of Center 1. Lastly, the final feature ranking was generated by averaging the 100-fold feature importance and normalizing them to the sum of 1.0. Features higher than half of the highest feature rank were considered high-ranking and were analyzed for imaging biomarker identifications.

For details of the automated machine learning process, including all methodological steps and their parameters, see the Supplemental Material.

### Independent Validation of the Prediction Model

The dataset of Center 2 was utilized as an independent test set to validate the established predictive model built by the dataset of Center 1. Confusion matrix analytics were utilized to calculate the number of true-positive, true-negative, false-positive, and false-negative prediction occurrences of each Center 2 case. Sensitivity, specificity, positive predictive value, accuracy, and area under the receiver operator characteristic (ROC) curve (AUC) were calculated across the validation cases.

## Results

### Patient Data

**Figure 1:**
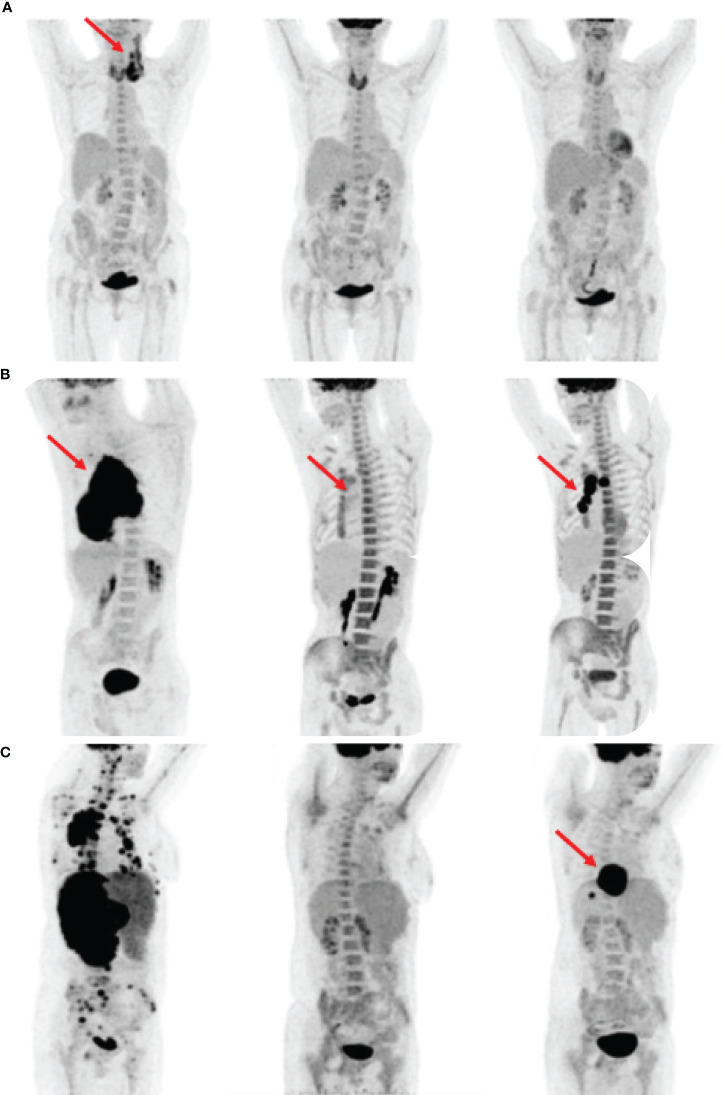
Comparison of clinical outcomes based on maximum intensity projection (MIP) images in three patients **(A–C)**. By each patient, the first image shows primary staging, the second shows interim PET scan, and the third shows post-treatment restaging scan. The red arrows indicate FDG avid lymphoma foci. **(A)** Patient in complete remission to treatment. The increased FDG uptake in all three images was a sign of thyroiditis. **(B)** Patient without complete remission during and after the therapy. The interim scan showed Deauville score 4. **(C)** Patient had an interim scan with Deauville score 3 but relapsed after the treatment.

At the end of the standard induction therapy, 55 patients achieved complete metabolic remission. During the 2-year follow-up, 14 patients had primary refractory disease, 14 patients relapsed within 12 months, and 2 patients had relapsed within 24 months. In summary, after the end of therapy, 30 patients had detectable metabolically active tumor tissue and relapsed within 24 months (**Figure 1**).

### Statistical Analysis

Using the data of the chi-square test, a significant association between COO, R-IPI, or stages and the specified groups was identified. There were significantly more patients in group 1 with non-GCB subtype, who had higher R-IPI values and stages. There was no significant difference between the sexes and between the groups. The clinicopathological features of patients are described in **Table 1**. In addition, patients were divided on the basis of the center where the [^18^F]FDG PET/CT scan was performed. No association between the two above-specified clinical outcomes (based on 2-year EFS), R-IPI, stages, or COO and the center where the examinations were performed was identified (**Table 2**).

**Table 1:** Comparison of clinical outcome of the patients and their clinical data.

Variables	No Progression or Remission	Progression within 24 months	*p*-value
**Sex, *n* = 85**	**(*n* = 55)**	**(*n* = 30)**	0.611
Male (*n*, %)	28 (32.9%)	17 (20%)	
Female (*n*, %)	27 (31.8%)	13 (15.3%)	
**ECOG, *n* = 83**	**(*n* = 55)**	**(*n* = 28)**	0.113
0 (*n*, %)	16 (19.3%)	6 (7.2%)	
1 (*n*, %)	26 (31.3%)	8 (9.6%)	
2 (*n*, %)	11 (13.3%)	12 (14.5%)	
3 (*n*, %)	2 (2.4%)	2 (2.4%)	
**Stage, *n* = 85**	**(*n* = 55)**	**(*n* = 30)**	0.017
1 (*n*, %)	10 (11.8%)	0	
2 (*n*, %)	17 (20%)	5 (5.9%)	
3 (*n*, %)	9 (10.6%)	8 (9.4%)	
4 (*n*, %)	19 (22.6%)	17 (20%)	
**R-IPI, *n* = 85**	**(*n* = 55)**	**(*n* = 30)**	0.015
0 (*n*, %)	7 (8.2%)	1 (1.2%)	
1 (*n*, %)	29 (34.1%)	9 (10.6%)	
2 (*n*, %)	19 (22.6%)	20 (23.5%)	
**COO, *n* = 82**	**(*n* = 53)**	**(*n* = 29)**	0.018
GC (*n*, %)	27 (32.9%)	7 (8.5%)	
N-GC (*n*, %)	26 (31.7%)	22 (26.8%)	

Chi-square test was performed in order to find the association between the outcome and the specified clinical status of the patients suffering from DLBCL.

**Table 2:** Comparison of patients regarding to the two clinical centers where the FDG PET/CT examinations were performed.

Variables	Center 1(Pécs)	Center 2 (Kaposvár)	*p*-value
	(*n* = 41)	(*n* = 44)	
**Clinical outcome, *n* = 85**			0.487
No Progression or Remission (*n*, %)	25 (29.4%)	30 (35.3%)	
Progression within 24 months (*n*, %)	16 (18.8%)	14 (16.5%)	
**Lymphoma stage, *n* = 85**			0.877
1 (*n*, %)	6 (7%)	4 (4.7%)
2 (*n*, %)	11 (12.9%)	11 (12.9%)
3 (*n*, %)	10 (11.8%)	7 (8.2%)
4 (*n*, %)	14 (16.5%)	22 (25.9%)
**R-IPI, *n* = 85**			0.988
0 (*n*, %)	4 (4.7%)	4 (4.7%)
1 (*n*, %)	18 (21.2%)	20 (23.5%)
2 (*n*, %)	19 (22.6%)	20 (23.5%)
**COO, *n* = 82**	**(*n* = 41)**	**(*n* = 41)**	0.654
GC (*n*, %)	18 (22%)	16 (19.5%)
N-GC (*n*, %)	23 (28%)	25 (30.5%)

### Automated Machine Learning Analysis and Biomarker Identification

Automated machine learning yielded 66% sensitivity, 77% specificity, 78% positive predictive value, 70% negative predictive value, 71% accuracy, and 0.74 AUC single-center cross-validation performance in Center 1.

**Figure 2:**
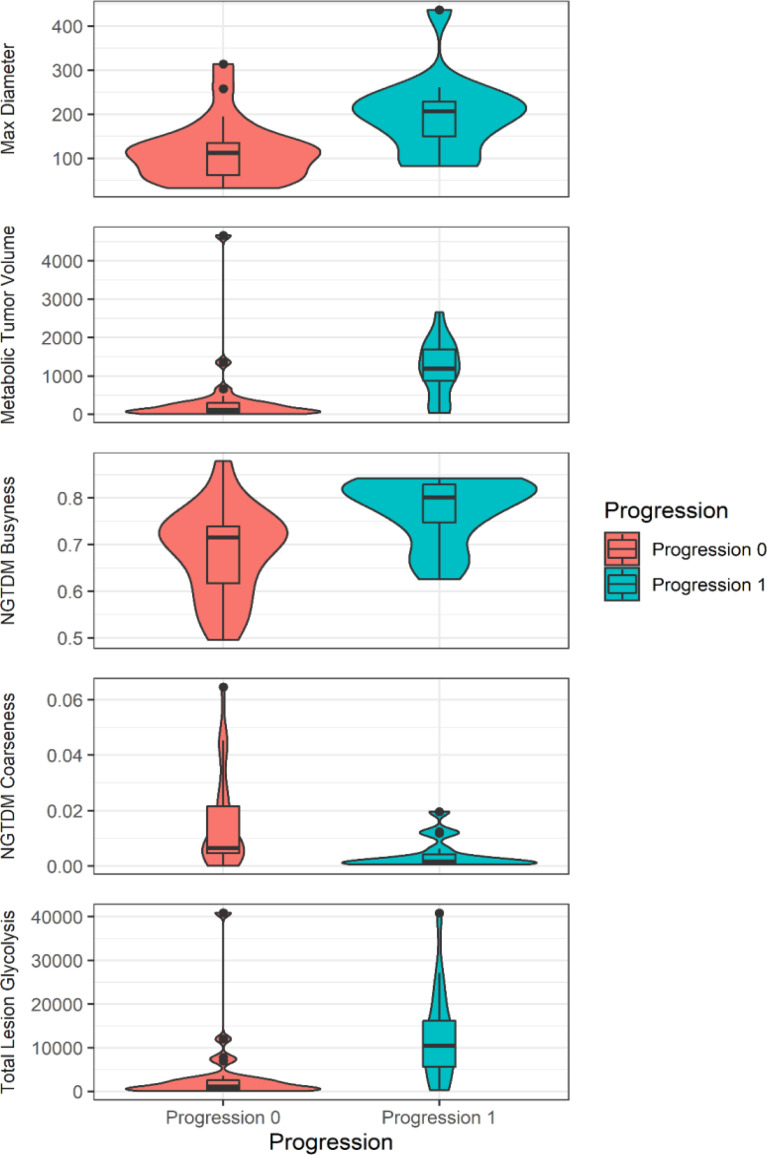
The violin plot (R: A Language and Environment for Statistical Computing, version 4.04., using package ggplot2, version 3.3.3) shows the values of the prominent features to predict 2-year event-free survival.

Feature ranking revealed that the most important features for building 2-year EFS prediction are as follows: max diameter (9%), NGTDM busyness (9%), TLG (8%), TMTV (8%), and NGTDM coarseness (5%). The distributions of these parameters are plotted on violin plots (**Figure 2**).

### Independent Validation of Prediction Model

**Figure 3:**
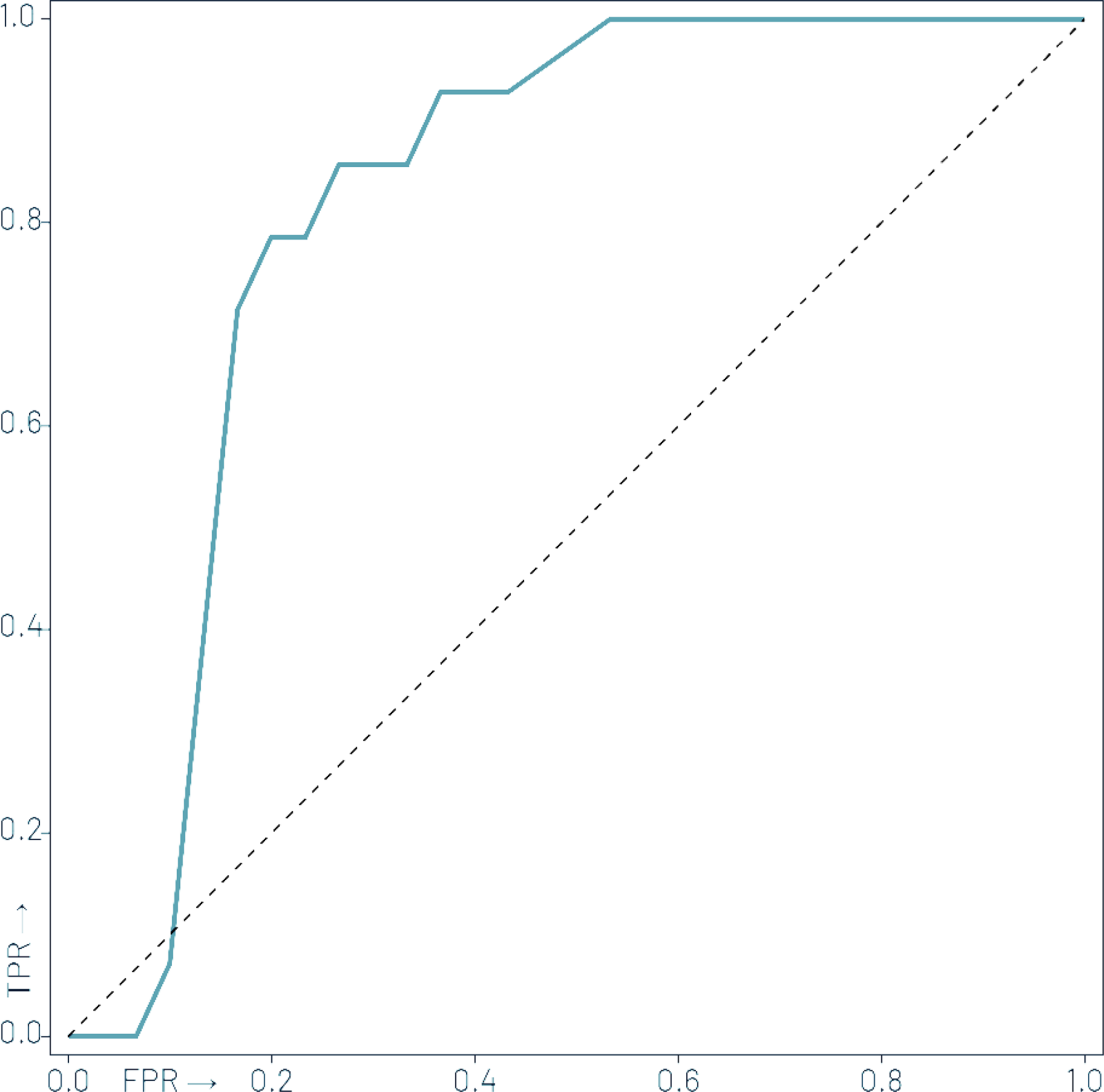
Receiver operator characteristic (ROC) curve of the independent validation performance of the machine learning model trained over Center 1 cases to predict 2-year event-free survival over Center 2 cases with an area under the ROC (AUC) of 0.85.

**Figure 4:**
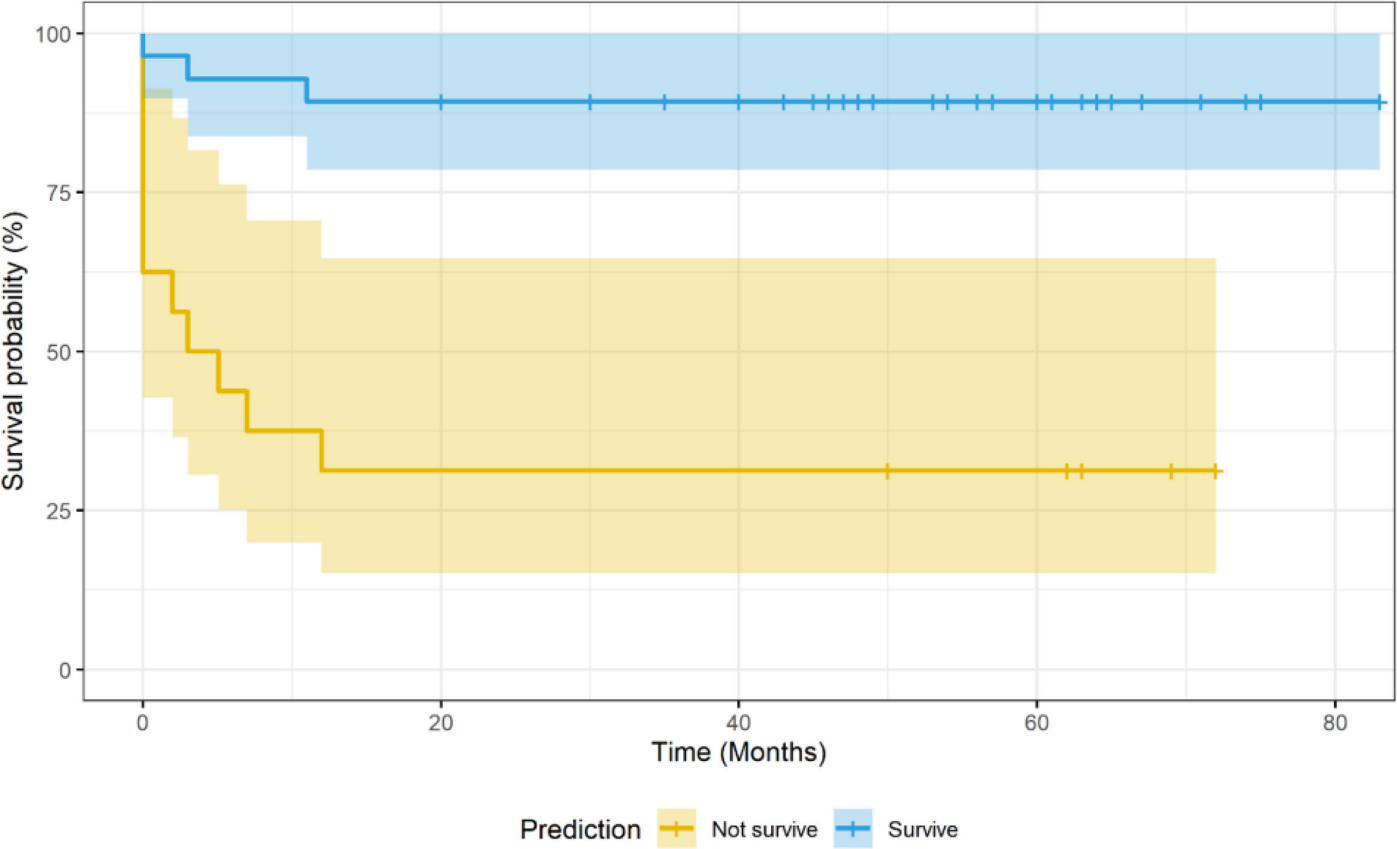
Kaplan–Meier curve of the machine learning (ML) model prediction vs. 2-year event-free survival in Center 2 cases. The ML model was trained with Center 1 cases.

The predictive model built on the Center 1 dataset yielded 79% sensitivity, 83% specificity, 69% positive predictive value, 89% negative predictive value, 82% accuracy, and 0.85 AUC by evaluating the Center 2 dataset. See **Figure 3** for the ROC curve of the independent validation performance over Center 2 cases. See **Figure 4** for the Kaplan–Meier curve of the machine learning prediction vs. EFS over samples of Center 2.

## Discussion

DLBCL is a heterogeneous disease at many levels with diverse genetic features and variable clinical outcomes (2, 7, 27). Although DLBCL is potentially curable with standard treatment, there is an urgent need for new therapies since most refractory or relapsed patients will eventually die from the disease. Based on available data, about 40% of patients either will be resistant to the initial line of therapy or will relapse after the initial response. The majority of these patients cannot be salvaged by high-dose chemotherapy followed by ASCT and eventually will succumb to their disease. A better understanding of the pathogenesis of disease could help us understand the unique characteristics and the course of different subtypes of disease. Tremendous progress has been made over the past 20 years to identify the subtypes of DLBCL based on the COO, which carry significant impact on the prognosis of patients. In 2000, Alizadeh et al. (28) performed gene expression profiling with cDNA microarrays to explore the molecular heterogeneity in DLBCL. They described at least two distinct groups within DLBCL: the GCB group and the activated B-cell-like (ABC) or non-GCB group. This method has been widely recognized as the first COO-based classification of DLBCL. In several randomized clinical trials following the establishment of COO classification by Alizadeh et al., DLBCL patients with the ABC subtype showed significantly poorer outcome compared with those with the GCB subtype, even when immune chemotherapy was used. In recent years, COO classification has been not only established as a prognostic factor but also used to target therapies for DLBCL patients. The World Health Organization (WHO) Classification for Lymphoid Malignancies requires the determination of COO for every newly diagnosed DLBCL case. In recent years, next-generation sequencing provides the possibility of more accurate classification of DLBCL. New DLBCL subgroups have been identified based on detailed molecular analysis, which may provide a more accurate prognosis prediction and pave the way for personalized target therapy (5, 7, 29).

In connection with these recent advances in the molecular classification of lymphoma, several international trials have examined whether pretreatment baseline PET or interim PET imaging can separate poor-responder patients requiring intensification of therapy from good responders to the standard treatment (30–33).

The prognostic classification of DLBCL patients was originally based on immunohistochemical and molecular genetic differences and laboratory and anamnestic data. In addition to these parameters, the results of [^18^F]FDG PET/CT have a strong and crucial prognostic significance. For the prognosis, the current evaluation of lymphoma and therapeutic efficacy in patients relies on Deauville scoring, using hepatic [^18^F]FDG uptake and mediastinal blood pool as reference value (11, 34).

To optimize therapy for outcomes, many recently published papers propose that in addition to the Deauville scoring and delta SUV-max, other semi-quantitative metabolic functional parameters from pretreatment functional imaging studies could be used including TMTV and TLG, which have been mostly studied in DLBCL (35–37).

In addition to these parameters, it would be important to measure tumor heterogeneity in lymphoma, which may also lead to a better prediction of prognosis. [^18^F]FDG PET/CT is one of these non-invasive methods that examine the intratumor metabolic heterogeneity at a macroscopic scale (19, 38). Many studies in different tumor types predicted additional prognostic outcomes from textural parameters describing tumor heterogeneity (39). Tumor heterogeneity in PET can be examined generally with the analysis of the histogram or the spatial arrangement of voxel intensities extracted by computational postprocessing techniques (40). These parameters have been intensively studied in DLBCL and in other tumor types and seem to be also useful to select high-risk patients, but no definitive clinical metric proposal has been formed yet (41–46).

We aimed to investigate the potential prognostic significance of metabolic heterogeneity descriptors derived from primary PET and compare their diagnostic value with conventional PET metrics, such as TMTV, TLG, and SUV-max, and clinical data using multicenter automated machine learning analysis. We hypothesized that we could identify and predict poor-responder patients, who may require additional molecular investigations, classification, and personalized, molecularly targeted treatment. For this, we retrospectively assessed the [^18^F]FDG PET scans of 85 patients, which were performed in 2 clinical centers. The predictive model built on data from the first center resulted in 79% sensitivity, 83% specificity, 69% positive predictive value, 89% negative predictive value, 82% accuracy, and an AUC of 0.85 on the second center dataset. Thus, based on clinical and imaging parameters determined before starting treatment, we were able to predict with high accuracy which patients would progress or relapse within 2 years of diagnosis. It is also important to point out that the cross-validation performance was better than within Center 1 performance, which implies high robustness and generalizability of the build model. It is important to emphasize that the independent validation performance was higher utilizing Center 2 than the within-Center 1 cross-validation performance. This has multiple reasons: On the one hand, Center 1 was further split into subsets to conduct the cross-validation, which also decreases predictability due to lower number of training subsets. On the other hand, the 100-fold Monte Carlo cross-validation scheme performs splitting randomly, which may result in training-validation subsets being less similar than the similarity of Center 1 and Center 2 that represent reality, instead of a simulated distribution.

Our analysis determined prominent features to predict 2-year EFS. Based on the applied feature ranking, three volume-based biomarkers (TMTV, TLG, and max diameter of the largest VOI) and two metabolic heterogeneity descriptors (NGTDM busyness and coarseness) had the highest diagnostic significance. Volume-based parameters refer to the extent of the lymphoma. The prognostic value of semi-quantitative metrics such as TMTV and TLG in lymphoma as well as in other tumor types has already been demonstrated (35–37). In our study, the max diameter of the largest lymphoma foci appears to be a better prognosis predictor than TMTV (see **Supplementary Material**: Feature importance). While these features may be redundant, their overall importance compared to each other may be different per cohort. Therefore, future investigations shall focus on identifying which of these two features are clinically relevant. While clinical parameters were included in our ML model building process, feature ranking did not select them as relevant for predicting 2-year survival, compared to imaging features. As such, the highest-ranking clinical feature was R-IPI with a ranking of 12 and with a relative importance of 2.53%. This implies that 2-year survival can be predicted with imaging features, which may act as surrogates of, albeit being superior to clinical parameters.

According to the IBSI “Textures with large changes in grey levels between neighboring voxels are said to be busy” (40). If the busyness is high, the neighboring uptake change is sudden and not smooth. The violin plot shows that group 1 with poor prognosis has higher busyness values. This may be explained by the fact that lymphoma cells are embedded in a necrotic, sometimes hypoxic, periphery, which may be a key point of the ineffectiveness of therapy, also because the chemotherapeutic agent may experience difficulty penetrating these regions. Furthermore, gray-level differences in coarse textures are generally small due to large-scale patterns. Summing differences gives an indication of the level of the spatial rate of change in intensity. This means that high coarseness is associated with larger regions in the lesions, while low coarseness indicates that the texture subregions are smaller. The violin plot shows that group 1 with poor prognosis has lower coarseness values. Coarseness can be associated with cell diversities within the volume regarding their different FDG uptake, which can be due to less proliferative tumor cells, or an inhomogeneous tumor mass with necrosis and hypoxic area.

In a study predicting 2-year EFS in DLBCL, low gray-level emphasis provides better prognosis prediction than TMTV, or coarseness and busyness (41), which we assume is due to population differences. Therefore, to determine the real importance of these features, prospective studies with more patients shall be performed.

In other—mostly solid—tumor types, coarseness was highlighted in predicting the outcome of locally advanced rectum cancer (45). In another study, both coarseness and busyness proved to be more predictive than other SUV-based parameters in non-small cell lung cancer (47).

Among SUV-based metrics, SUV-max is the most used parameter in routine diagnostics. With SUV-max, indolent and aggressive lymphomas could be well-differentiated, and this metric is also correlated with tumor histology (proliferation rate) and blood levels of enzymes, for example, KI-67 status and LDH (48). Several research groups have already demonstrated the diagnostic value of SUV-max in lymphoma during primer staging; in one of them, SUV-max proved to be more prognostic than TMTV or TLG (49). However, in our study, these parameters were less important features than others. We hypothesized that the SUV measurements are more influenced by the instrumentations and environmental factors than volume-based and textural parameters.

In addition to PET parameters, clinical parameters are also crucial in the prediction of prognosis, and this fact was confirmed by our statistical approach even if these parameters had lower ranks than some PET parameters. Using chi-square test results, we found a significant relationship between the DLBCL subtype groups and clinical and pathological parameters such as R-IPI and COO. Patients with non-GCB or higher R-IPI values have a significantly worse 2-year prognosis as reported in many previous studies. The prognostic value and diagnostic significance of COO and R-IPI have been known for a very long time (4, 28). The COO can be easily determined in all patients, mainly according to the Hans algorithm, and its combination with TMTV has been suggested by some studies (50). R-IPI proved to be more prognostic than TMTV in another study (51). However, most of the studies use one or a maximum of two metrics for prognosis assessment (50, 52). In contrast, machine learning-built prediction models have the potential to deliver more in-depth associations among clinical and PET data (53–55).

This study had limitations. First, we analyzed only the largest VOI in each patient with radiomics. Nevertheless, prior studies routinely analyzed the largest VOIs in DLBCL patients and yielded promising results (41, 44). In addition, radiomic analysis is generally discouraged to be performed in small lesions. Second, our patient counts were relatively low in both Center 1 and Center 2 cohorts. Nevertheless, they were from different camera systems, which allowed us to perform an independent validation scheme of our predictive model.

With our dual-center study, we could demonstrate that predicting 2-year progression-free survival in DLBCL patients is feasible with high-precision building on imaging and clinical parameters. This is in line with prior studies that utilize holistic datasets to build so-called holomics prediction models with machine learning (16, 25, 56). Given that our model yielded a balanced sensitivity and specificity, it could be a viable option to personalize the patient’s treatment. In the era of personalized medicine, with more detailed and specialized molecular diagnostics—especially in DLBCL—this could help clinicians to manage their patients more adequately and effectively.

## Conclusion

Based on our dual-center retrospective analysis, predicting 2-year EFS built on imaging features is feasible by utilizing high-performance automated machine learning. Subsequent DLBCL studies shall further evaluate the identified imaging biomarkers and their predictive performance in other clinical settings.

## Data Availability Statement

The original contributions presented in the study are included in the article/**Supplementary Material**. Further inquiries can be directed to the corresponding author.

## Ethics Statement

The studies involving human participants were reviewed and approved by the appropriate local institutional research ethics committee and the Hungarian National Institute of Pharmacy and Nutrition under permission number 6536 – University of Pécs 2017 and OGYÉI/50268-8/2017. Written informed consent for participation was not required for this study in accordance with the national legislation and the institutional requirements.

## Author Contributions

Conceptualization: ZR, KZ, and HA. Methodology: LPapp and DM. Software: LPapp. Formal analysis: FB and ES. Investigation: DD and ZR. Resources: DM and ES. Data curation: ZT, LPaj, AB, ÉK, and ÁS. Writing—original draft preparation: ZR and HA. Writing—review and editing: LPapp, HA, KZ, and ES. Visualization: DM and DV. Supervision: HA, KZ, and LPapp. Project administration: ES and ZR. Funding acquisition: HA, DM, and KZ. All authors commented on previous versions of the manuscript. All authors have read and agreed to the published version of the manuscript.

## Funding

The research was supported by the Hungarian Government`s higher Education Institutional Excellence Program project 20765-3/2018/FEKUTSTRAT. Research leading to these results has received funding from the European Union’s Horizon 2020 research and innovation program under grant agreement No. 739593. HCEMM was supported by EU Program: H2020-EU.4.a. This work was also partly funded by a grant from the Hungarian National Research, Development and Innovation Office (Thematic Excellence Program, TKP-BIOImaging, financed under the 2020-4.1.1-TKP2020 funding scheme).

## Conflict of Interest

The authors declare that the research was conducted in the absence of any commercial or financial relationships that could be construed as a potential conflict of interest.

## Publisher’s Note

All claims expressed in this article are solely those of the authors and do not necessarily represent those of their affiliated organizations, or those of the publisher, the editors and the reviewers. Any product that may be evaluated in this article, or claim that may be made by its manufacturer, is not guaranteed or endorsed by the publisher.
