# Supplementary material for: Two-Year Event-Free Survival Prediction in DLBCL Patients Based on In Vivo Radiomics and Clinical Parameters
Source: Front Oncol. 2022 Jun 8;12:820136. doi: 10.3389/fonc.2022.820136 (PMC9216187; doi:10.3389/fonc.2022.820136)
Supplement: Supplementary file 1 [file DataSheet_1.docx]

# Supplemental Material

**Two-year event-free survival prediction in DLBCL patients based on *in vivo* radiomics and clinical parameters**

**RADIOMIC FEATURE EXTRACTION**

**Table 1.** the Imaging Biomarker Standardization Initiative (IBSI) reporting structure of the study. The information presented herein is based on the IBSI guidelines (1).

| **Patient** | | |
| --- | --- | --- |
| Volume of Interest | Predictive value of pretreatment [18F]FDG PET in DLBCL | |
| Patient Preparation | Blood glucose level <8 mmol/l  24 h inactivity and 6 h fasting before tracer administration | |
| Radiotracer | [18F]FDG PET | Fluorodeoxyglucose |
| **Acquisition and Reconstruction** | | |
| Protocol | Intravenous injection of [18F]FDG 3-4 MBq/kg  60 minutes relaxation after tracer administration  No speaking, chewing, or reading was allowed. | |
| Scanner type | Center 1: AnyScan PET/CT (Mediso Medical Imaging Systems)  Center 2: Siemens Biograph Truepoint (Siemens) | |
| [18F]FDG PET in Center 1 | 3D acquisition method  Frame time: 3 minutes  Bed positions: 7-10  Axial FOV: 15,12 cm  Tera-Tomo™ 3D image reconstruction algorithm  Matrix: 167x167  Voxel size: 4 mm. | |
| [18F]FDG PET in Center 2 | 3D acquisition method  Frame time: 3 minutes  Bed positions: 7-9  Axial FOV:16,2 cm  2D OSEM (3i8s, 5 mm Gaussian filtering) image reconstruction algorithm  Matrix: 168x168  Voxel size: 5 mm | |
| CT in Center 1 | X-Ray tube voltage: 120kVp (depending on the patient’s size, 140kVp is used in bariatric patients),  X-Ray tube current: 24–26 mAs (also depending on the patients’ size, higher tube current can be applied in bariatric patient).  Pitch: 1,5  Slice thickness: 2,5 mm | |
| CT in Center 2 | X-Ray tube voltage: 120kVp (depending on the patient’s size, 140kVp is used in bariatric patients),  X-Ray tube current: Reference effective mAs:60 using CareDose  Pitch: 1,5  Slice thickness: 5 mm | |
| **Data conversion** | | |
| Step 1 | BQML voxel units were transformed to weight-normalized SUV automatically by the Interview FUSION software (Mediso). | |
| **Segmentation** | | |
| Software | Interview FUSION ver 3.10 | |
| VOI definition | Standard semi-automated iso-count 3D. | |
| Number of experts | 1+1 (1 nuclear medicine expert participated in independent delineations, followed by 1 senior nuclear medicine specialist cross-validation and if necessary, modification of first-round results) | |
| Reference image | PET | |
| **Image / VOI interpolation** | | |
| Method | Cubic 3D, including nearest 26 neighbors | |
| Grid | Align by center | |
| Extrapolation beyond original image | Neighbor distance search calculated as original voxel size main diagonal + epsilon. Missing value: image minimum | |
| Voxel dimensions | 4.0 x 4.0 x 4.0 mm | |
| Partially masked voxels (VOI) | Taken if more than half of original voxel area included | |
| **Discretization** | | |
| Method | Fixed bin width, variable number of bins | |
| Bin width | - 0.1 SUV | |
| **Image biomarker computation / Parameters** | | |
| **Intensity features** (6): Minimum, Maximum, Mean, Sum, Variance, Local intensity peak  **Histogram features** (2): Discretised intensity skewness, (Excess) discretised intensity kurtosis  **GLCM features** (4): Joint entropy, Contrast, Inverse difference, Correlation  **Morphological features** (2): Volume (mesh), Maximum 3D diameter  **GLSZM features** (12): Small zone emphasis, Large zone emphasis, Low grey level zone emphasis, High grey level zone emphasis, Small zone low grey level emphasis, Small zone high grey level emphasis, Large zone low grey level emphasis, Large zone high grey level emphasis, Grey level non-uniformity, Zone size non-uniformity, Zone percentage, Zone size variance  **NGTDM features** (3): Coarseness, Busyness, Complexity  **GLRLM features** (11): Short runs emphasis, Long runs emphasis, Low grey level run emphasis, High grey level run emphasis, Short run low grey level emphasis, Short run high grey level emphasis, Long run low grey level emphasis, Long run high grey level emphasis, Grey level non-uniformity, Run length non-uniformity, Run percentage | | |
| Software | Interview FUSION ver. 3.10 | |
| Distance weighting | No | |
| CM symmetry | Symmetric | |
| CM / ZM distance | Chebyshev distance 1 | |
| CM / ZM aggregation | 3D, full-merging | |
| Exclusion criteria | VOIs with less than 64 voxels were excluded from the analysis | |

## Automated Machine Learning Analysis

Tabular data submission for Dedicaid AutoML services was performed by Dedicaid user laszlo.papp@meduniwien.ac.at on 5/7/2021, 9:10:06 AM to build and cross-validate automated data preprocessing and mixed, stacked ensemble learning pipelines for predicting reference label Prog_24_months. For the details of the analysis see Table 2.

**Table 2.** Properties of the automated machine learning (AutoML) analysis of this study.

| Data name | PECS-DLBCL with the largest VOI.xlsx |
| --- | --- |
| Data size | 41 samples, 57 features |
| Date of analysis | 5/7/2021, 9:10:06 AM |
| Duration of analysis | 20h 58m 46s |
| Submitted by | laszlo.papp@meduniwien.ac.at |
| Dedicaid AutoML version | 1.0 (MUW offline) |

## Methods

### Data

The input dataset was composed of 41 samples and 57 features. The submitted dataset was composed of 57 features. The selected reference label for the cross-validation was Prog_24_months having subgroups of 0 (60.9%) and 1 (39%) label outcomes.

### Cross-Validation

Monte Carlo (MC) cross-validation scheme was applied with 80% training and 20% validation ratios across 100 folds (3). Each fold had unique training-validation configurations. MC split resulted in 33 samples per fold in the training set. The validation set of each fold contained 4 samples per reference label (8 overall). The validation samples were equally subsampled to ensure that none of the label outcomes are over or underrepresented during the cross-validation.

### Preprocessing

The data underwent preprocessing steps in each fold before performing machine learning (ML) analysis. Preprocessing resulted in average 102 samples and 13 features across all MC folds. For the preprocessing steps and their parameters, see Table 3.

**Table 3.** Preprocessing step algorithms as well as their parameter values performed in all Monte Carlo folds before machine learning. FN - Feature Normalization; SRR - Smart Redundancy Reduction; SSYN - Sample Synthetizer.

| Preprocessing step | Algorithm | Parameter | Value | Reference |
| --- | --- | --- | --- | --- |
| 1 | FN | Normalization type | Mean-Deviation | (4) |
| 2 | SRR | Redundancy Threshold (Covariance) | 0.85 | (5) |
| 3 | SSYN | Oversampling ratio (majority subgroup) | 2.32 | (6) |
|  |  | Sampling technique | SMOTE |  |

### Machine Learning Layer 1

Various machine learning algorithms were established in each fold to minimize the effect of algorithm bias (5). Each model was trained by randomly selecting 80% of the preprocessed training data per MC fold. For details of the ML algorithms, see Table 4.

**Table 4.** Machine learning (ML) algorithms in the first ML layer with their parameters and value ranges across Monte Carlo (MC) folds. Occurrence of each ML type is represented in percentages across MC folds. BYS – Bayesian Classifier; MGWC – Multi-Gaussian Weighted Classifier; RF – Random Forest Classifier; SVM – Support Vector Machine Classifier; MLP – Multi-Layer Perceptron Classifier;

| ML Algorithm | Parameter | Value Range | Occurrence | Reference |
| --- | --- | --- | --- | --- |
| BYS | – | – | 23.86% | (7) |
| MGWC | Initial value multiplier | 1 – 10 | 23.86% | (8) |
|  | Maximum iterations | 13000 – 65000 |  |  |
|  | Negative weights allowed | false, true |  |  |
|  | Scale value multiplier | 0.1 – 50 |  |  |
|  | Tolerance | 0.00001 – 0.0001 |  |  |
| RF | Bag fraction | 0.8 – 0.9 | 23.86% | (5) |
|  | Bagging method | normal, equalized |  |  |
|  | Boosting | none, adaboost |  |  |
|  | Maximum tree depth | 5 – 28 |  |  |
|  | Minimum samples in leaves | 2 – 5 |  |  |
|  | Node feature selection method | none |  |  |
|  | Number of random features per node | 5 |  |  |
|  | Number of selected trees | 101 – 201 |  |  |
|  | Number of trees to build | 301 – 1001 |  |  |
|  | Tree quality metric | gain, gini |  |  |
|  | Tree selection method | 0 |  |  |
| SVM | Learning rate | 0.001 – 0.01 | 23.86% | (9) |
|  | Maximum iterations | 1000 – 5000 |  |  |
| MLP | Batch size ratio | 0.7 – 0.9 | 4.53% | (10) |
|  | Beta 1 | 0.9 |  |  |
|  | Beta 2 | 0.99 |  |  |
|  | Descent method | adam, vanilla |  |  |
|  | Dropout hidden layer probability | 0.1 – 0.2 |  |  |
|  | Dropout input layer probability | 0.1 – 0.2 |  |  |
|  | Learning rate | 0.001 – 0.01 |  |  |
|  | Loss method | EntropyLoss |  |  |
|  | Maximum epochs | 205 – 615 |  |  |
|  | Regularization method | Dropout, L2, L1 |  |  |
|  | Regularization parameter | 0.001 – 0.01 |  |  |

### Machine Learning Layer 2

Meta-training sets were created by evaluating the samples of the preprocessed training set in each MC fold by the trained models in ML layer 1. In order to create the meta-training set, the prediction results of each trained model in ML layer 1 were handled as feature values of the given training sample. The meta-training set was the input for training the second ML layer prediction models. These models were trained to identify patterns in the prediction of the first ML layer models to result in mixed super learners (11). For the parameters of the second layer ML algorithms see Table 5.

**Table 5.** Machine learning (ML) algorithms in the second ML layer with their parameters and value ranges across Monte Carlo (MC) folds. Occurrence of each ML type is represented in percentages across MC folds. MGWC – Multi-Gaussian Weighted Classifier; RF – Random Forest Classifier; SVM – Support Vector Machine Classifier;

| ML Algorithm | Parameter | Value Range | Occurrence | Reference |
| --- | --- | --- | --- | --- |
| MGWC | Initial value multiplier | 10 | 33.33% | (8) |
|  | Maximum iterations | 4000 – 5000 |  |  |
|  | Negative weights allowed | false, true |  |  |
|  | Scale value multiplier | 1 – 5 |  |  |
|  | Tolerance | 0.0001 |  |  |
| RF | Bag fraction | 0.8 – 0.99 | 33.33% | (5) |
|  | Bagging method | normal, equalized |  |  |
|  | Boosting | none, adaboost |  |  |
|  | Maximum tree depth | 5 |  |  |
|  | Minimum samples in leaves | 2 – 5 |  |  |
|  | Node feature selection method | none |  |  |
|  | Number of random features per node | 5 |  |  |
|  | Number of selected trees | 201 |  |  |
|  | Number of trees to build | 501 – 1001 |  |  |
|  | Tree quality metric | gini, gain |  |  |
|  | Tree selection method | 0 |  |  |
| SVM | Learning rate | 0.001 – 0.01 | 33.33% | (9) |
|  | Maximum iterations | 1000 – 5000 |  |  |

### Top-Layer Model

Combination of the prediction results of the second layer ML models was performed by weighted majority voting to provide the final prediction of the model scheme. Weighting of each ML Layer 2 model was calculated based on training performance. In addition, ML Layer 2 models having less training performance than the median of all ML layer 2 model training performances had weight 0 in the final vote.

## Results

### Cross-Validation Performance

Model prediction performance was estimated via the MC cross-validation scheme utilizing confusion matrix analytics (12). True positive, true negative, false positive and false negative confusion matrix entries were calculated by evaluating the validation samples by the established model pipeline in each fold. Sensitivity, specificity, accuracy, positive predictive as well as negative predictive values were calculated across the MC fold validation results. For the average cross-validation performance of ML Layer 1 and 2 models see Table 6 and Table 7 respectively. For the cross-validation results of the final (top-layer) prediction models as well as for the summary of the evaluation, see Table 8 and Figure 1. For cross-validation area under the curve (AUC), see Figure 2.

**Table 6.** Average Monte Carlo (MC) cross-validation performance (%) of ML Layer 1 (ML-1) predictive models as determined by confusion matrix analytics across all MC folds. BYS – Bayesian Classifier; MGWC – Multi-Gaussian Weighted Classifier; RF – Random Forest Classifier; SVM – Support Vector Machine Classifier; MLP – Multi-Layer Perceptron Classifier; SNS – Sensitivity; SPC – Specificity; PPV – Positive Predictive Value; NPV – Negative Predictive Value; ACC – Accuracy; OCC – Occurrence. Performance and occurrence values are in percentages.

|  | SNS | SPC | PPV | NPV | ACC | OCC |
| --- | --- | --- | --- | --- | --- | --- |
| BYS | 63 | 68 | 69 | 67 | 65 | 24 |
| MGWC | 52 | 85 | 70 | 66 | 68 | 24 |
| RF | 58 | 81 | 74 | 67 | 69 | 24 |
| SVM | 79 | 64 | 72 | 75 | 71 | 24 |
| MLP | 67 | 60 | 66 | 54 | 63 | 5 |

**Table 7.** Average Monte Carlo (MC) cross-validation performance (%) of ML Layer 2 (ML-2) predictive models as determined by confusion matrix analytics across all MC folds. MGWC – Multi-Gaussian Weighted Classifier; RF – Random Forest Classifier; SVM – Support Vector Machine Classifier; SNS – Sensitivity; SPC – Specificity; PPV – Positive Predictive Value; NPV – Negative Predictive Value; ACC – Accuracy; OCC – Occurrence. Performance and occurrence values are in percentages.

|  | SNS | SPC | PPV | NPV | ACC | OCC |
| --- | --- | --- | --- | --- | --- | --- |
| MGWC | 74 | 72 | 77 | 76 | 73 | 33 |
| RF | 65 | 77 | 77 | 70 | 71 | 33 |
| SVM | 75 | 66 | 72 | 75 | 70 | 33 |

**Table 8.** Performance Monte Carlo (MC) cross-validation performance of the established model scheme throughout the performance of the top-layer prediction model. Performance values were determined by confusion matrix analytics across all MC folds. MGWC – Multi-Gaussian Weighted Classifier; RF – Random Forest Classifier; SVM – Support Vector Machine Classifier; SNS – Sensitivity; SPC – Specificity; PPV – Positive Predictive Value; NPV – Negative Predictive Value; ACC – Accuracy; AUC – Area Under the Receiver Operator Characteristics Curve. Performance values are in percentages. LQ – Lower quartile; UQ – Upper Quartile; Dev – Mean Deviation.

|  | Min | LQ | Median | UQ | Max | Mean | Dev |
| --- | --- | --- | --- | --- | --- | --- | --- |
| SNS | 0 | 33.33 | 66.66 | 100 | 100 | 65.66 | 19.46 |
| SPC | 0 | 66.66 | 83.33 | 100 | 100 | 76.66 | 23.33 |
| PPV | 0 | 60 | 75 | 100 | 100 | 77.43 | 21.56 |
| NPV | 0 | 60 | 75 | 100 | 100 | 69.93 | 17.53 |
| ACC | 33.33 | 66.66 | 66.66 | 83.33 | 100 | 71.16 | 15.19 |
| AUC | 33.33 | 61.11 | 77.77 | 83.33 | 100 | 74.33 | 14.96 |


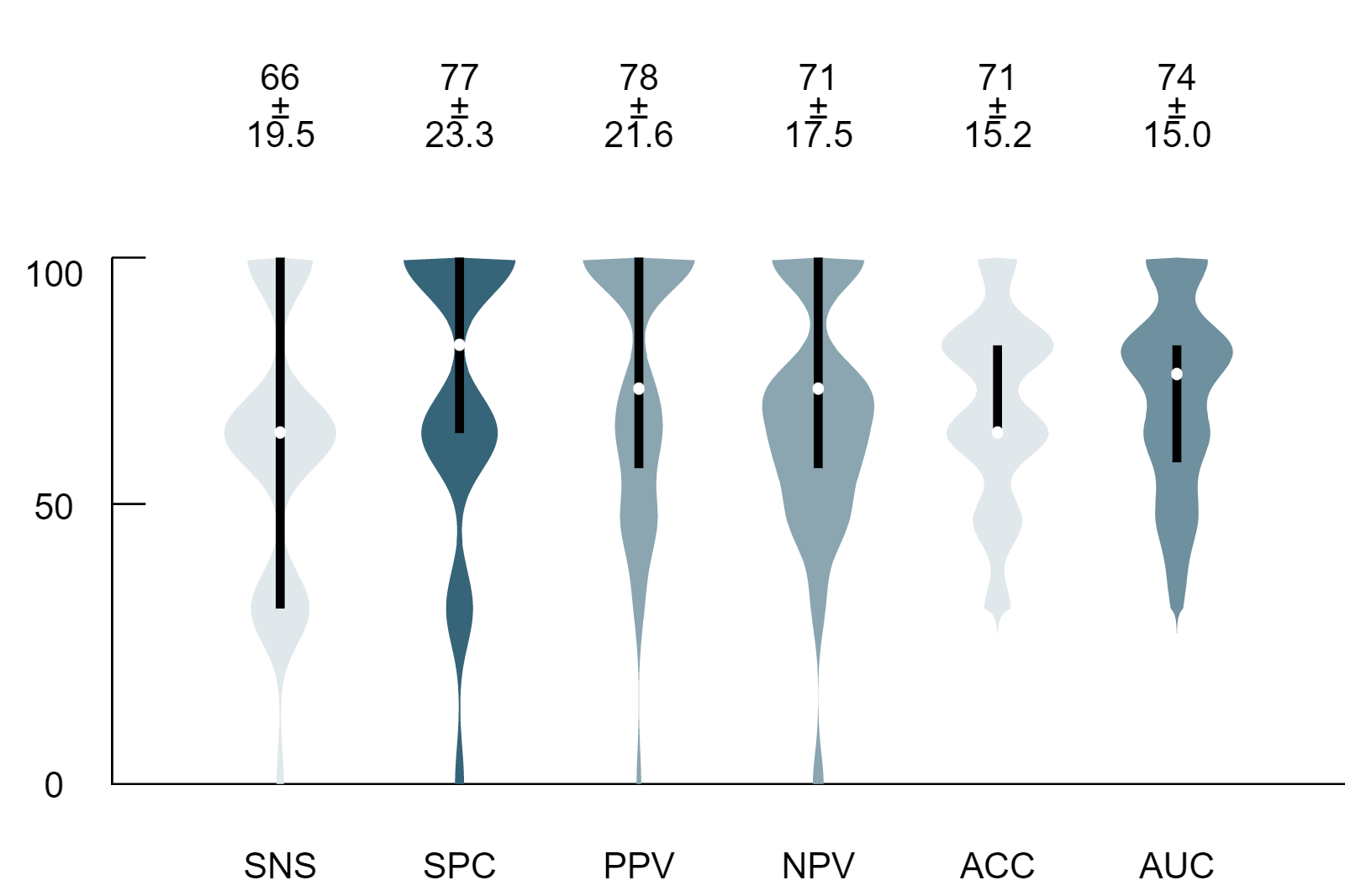


**Figure 1.** Box-plot Monte Carlo (MC) cross-validation performance of the established model scheme throughout the performance of the top-layer prediction model. Performance values were determined by confusion matrix analytics across all MC folds. SNS – Sensitivity; SPC – Specificity; PPV – Positive Predictive Value; NPV – Negative Predictive Value; ACC – Accuracy; AUC – Area Under the Receiver Operator Characteristics Curve. Performance values are in percentages.


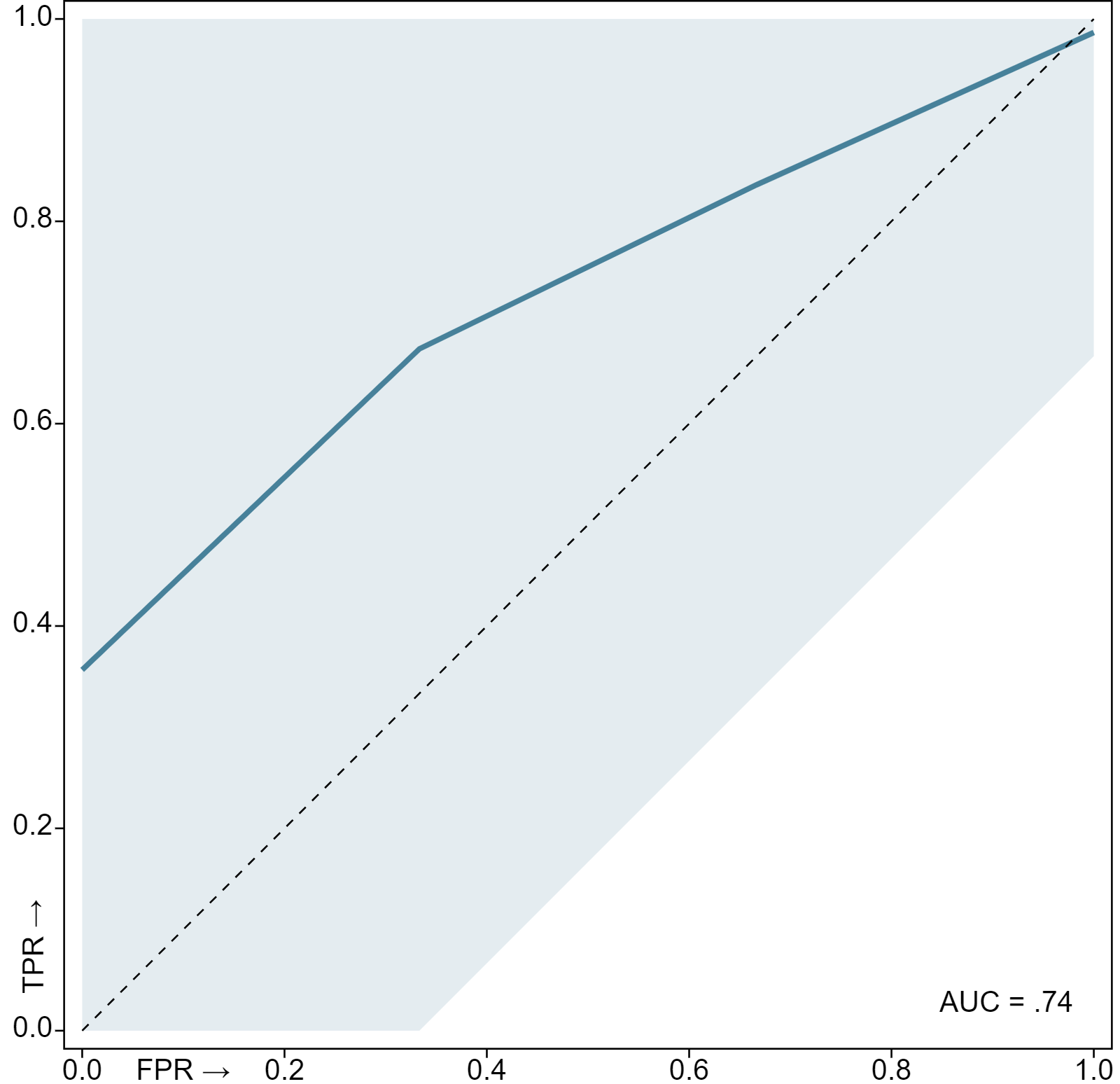


**Figure 2.** Mean cross-validation Receiver Operator Characteristics (ROC) curve of the built models. The thick blue line corresponds to the mean ROC curve, while the light blue shaded area represents the spread of all 100 ROC curves generated across the validation folds. AUC – Area Under the Receiver Operator Characteristics Curve (mean of all 100 AUCs); FPR – False Positive Rate, TPR – True Positive Rate. Dashed diagonal line represents a reference random-guess AUC for comparison.

### Feature Importance

Feature ranking and selection was performed as part of the data preprocessing steps of each fold (see Sec. Preprocessing). The final feature importance was calculated as the mean of all feature rankings across the MC folds. Note that only the highest-ranking 30 features are shown in Table 9.

**Table 9.** Selected features and their ranks as calculated across the MC folds by Smart Redundancy Reduction (SRR - see Table 2) as well as their respective value distributions. Ranks represent the relative importance of selected features for model building. Features are ordered by ranks. Rank values are in percentages. LQ – Lower quartile; UQ – Upper Quartile; Dev – Mean Deviation.

| Feature Name | Mean Ranking ±Dev | Min | LQ | Median | UQ | Max |
| --- | --- | --- | --- | --- | --- | --- |
|  |  | Feature Value Histogram | | | | |
| Max_Diameter_mm_VOI | 8.49% ±1.01% | 5.74% | 7.63% | 8.53% | 9.32% | 11.58% |
|  |  | 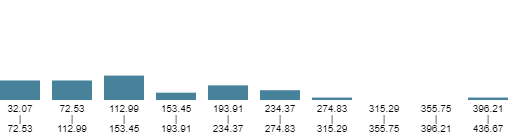 | | | | |
| Busyness | 7.75% ±0.91% | 5.52% | 6.85% | 7.96% | 8.49% | 10.31% |
|  |  | 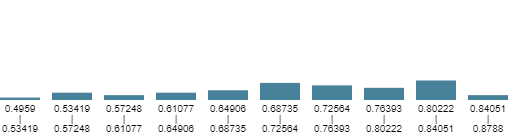 | | | | |
| TLG_g_Total | 6.85% ±0.74% | 5.21% | 6.09% | 6.76% | 7.51% | 9.98% |
|  |  | 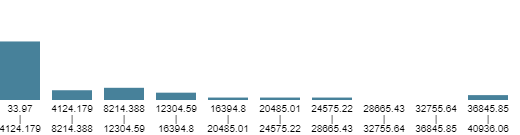 | | | | |
| MTV_cm3 | 6.27% ±0.93% | 4.34% | 5.36% | 6.04% | 6.4% | 12.05% |
|  |  | 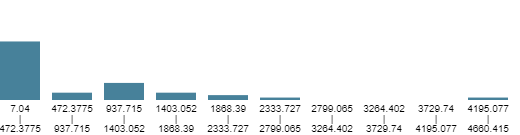 | | | | |
| Coarseness | 5.03% ±0.73% | 3.1% | 4.51% | 4.99% | 5.65% | 7.86% |
|  |  | 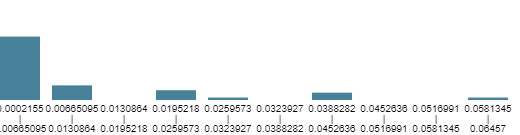 | | | | |
| Long_Run_High_Grey_Level_Empha sis | 4.07% ±0.95% | 1.6% | 3.12% | 4.19% | 4.87% | 7.41% |
|  |  | 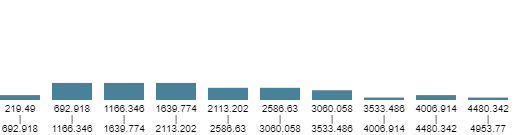 | | | | |
| Sum | 4.03% ±0.77% | 1.87% | 3.26% | 3.91% | 4.59% | 6.92% |
|  |  | 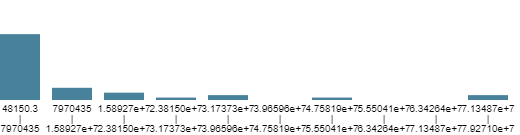 | | | | |
| TLG_VOI | 3.74% ±0.78% | 1.52% | 3.02% | 3.65% | 4.33% | 6.56% |
|  |  | 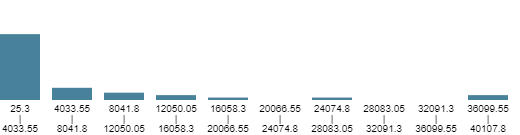 | | | | |
| Correlation | 3.47% ±0.77% | 1.61% | 2.73% | 3.42% | 4.12% | 5.68% |
|  |  | 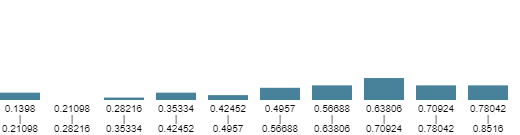 | | | | |
| No_Pixels_Voxels | 3.02% ±0.95% | 1.45% | 2.26% | 2.61% | 3.21% | 8.65% |
|  |  | 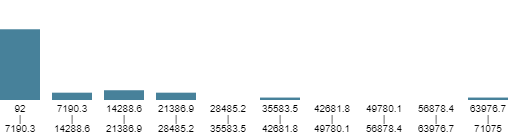 | | | | |
| HardArea_Volume | 3.01% ±0.95% | 1.45% | 2.26% | 2.6% | 3.2% | 8.64% |
|  |  | 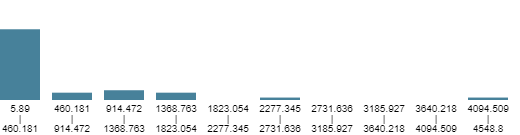 | | | | |
| R_IPI | 2.53% ±0.82% | 0.46% | 1.8% | 2.46% | 3.29% | 5.68% |
|  |  | 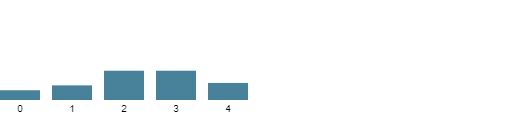 | | | | |
| Complexity | 2.38% ±0.91% | 0.63% | 1.46% | 2.25% | 3.16% | 5.64% |
|  |  | 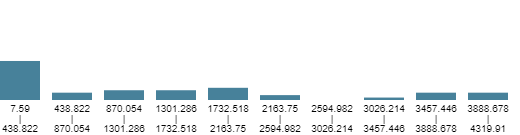 | | | | |
| ECOG | 2.36% ±0.9% | 0.25% | 1.5% | 2.3% | 2.96% | 5.41% |
|  |  | 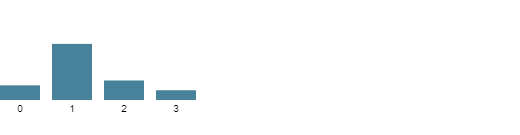 | | | | |
| Lymphoma_Stage | 2.3% ±0.71% | 0.66% | 1.67% | 2.25% | 2.8% | 5.62% |
|  |  | 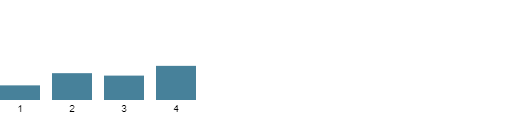 | | | | |
| Min | 2.21% ±1.02% | 0.1% | 1.28% | 1.92% | 3.01% | 5.99% |
|  |  | 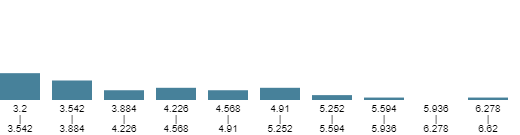 | | | | |
| Skewness | 2.03% ±0.77% | 0.32% | 1.3% | 1.91% | 2.52% | 4.58% |
|  |  | 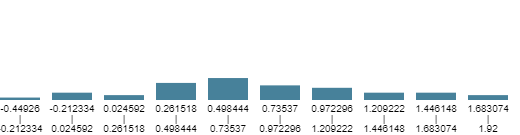 | | | | |
| Short_Run_Low_Grey_Level_Empha sis | 1.84% ±0.36% | 0.94% | 1.48% | 1.84% | 2.13% | 2.97% |
|  |  | 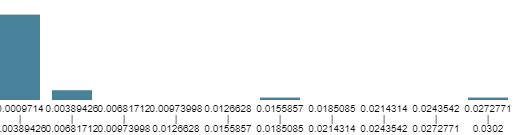 | | | | |
| COO_GC0_NGC1 | 1.79% ±0.89% | 0.2% | 0.88% | 1.59% | 2.5% | 5.23% |
|  |  | 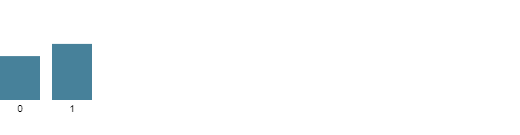 | | | | |
| Long_Zone_High_Grey_Level_Emph asis | 1.37% ±0.54% | 0% | 1.15% | 1.56% | 1.78% | 2.84% |
|  |  | 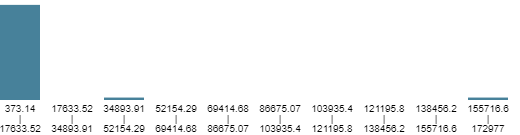 | | | | |
| Low_Grey_Level_Run_Emphasis | 1.36% ±0.29% | 0.67% | 1.09% | 1.34% | 1.58% | 2.24% |
|  |  | 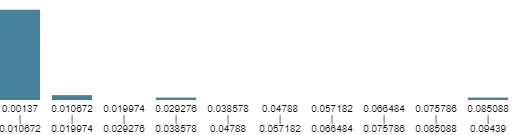 | | | | |
| Contrast | 1.33% ±0.53% | 0.12% | 0.87% | 1.16% | 1.67% | 3.18% |
|  |  | 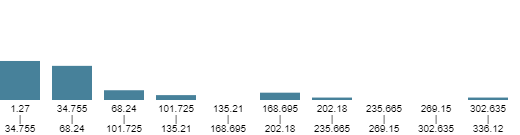 | | | | |
| Low_Grey_Level_Zone_Emphasis | 1.29% ±0.26% | 0.63% | 1.05% | 1.28% | 1.49% | 2.13% |
|  |  | 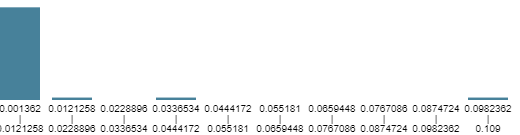 | | | | |
| Kurtosis | 1.25% ±0.73% | 0.03% | 0.55% | 0.98% | 1.67% | 4.45% |
|  |  | 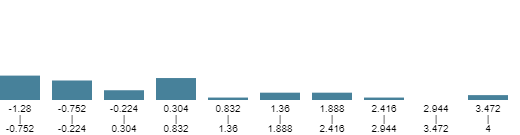 | | | | |
| Short_Zone_Low_Grey_Level_Emph asis | 1.24% ±0.24% | 0.59% | 1.01% | 1.21% | 1.43% | 1.97% |
|  |  | 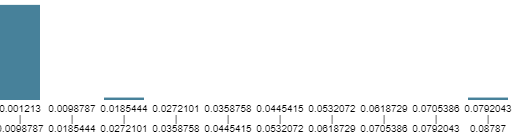 | | | | |
| Run_Length_Non_Uniformity | 1.13% ±0.53% | 0% | 0.66% | 1.07% | 1.52% | 2.81% |
|  |  | 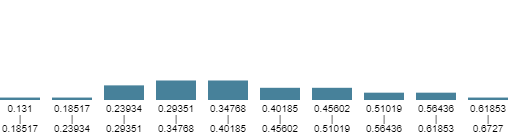 | | | | |
| Gray_Level_Non_Uniformity_A | 1.13% ±0.36% | 0.28% | 0.84% | 1.11% | 1.43% | 2.43% |
|  |  | 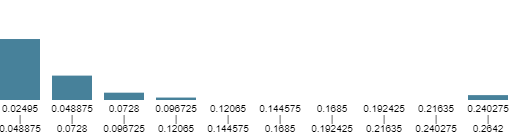 | | | | |
| Size_Var | 1.1% ±0.39% | 0.18% | 0.73% | 1.04% | 1.41% | 2.59% |
|  |  | 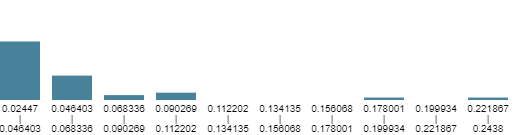 | | | | |
| Gray_Level_Non_Uniformity | 1.1% ±0.39% | 0.18% | 0.73% | 1.04% | 1.41% | 2.59% |
|  |  | 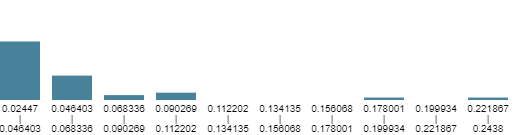 | | | | |
| R_IPI_2 | 1.01% ±0.57% | 0.01% | 0.42% | 0.91% | 1.44% | 3.18% |
|  |  | 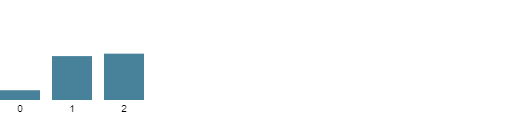 | | | | |

### REFERENCES

1. Zwanenburg A, Leger S, Vallières M, Löck S, Initiative for the IBS. Image biomarker standardisation initiative. arXiv [Internet]. 2016;(November). Available from: http://arxiv.org/abs/1612.07003

2. van der Maaten LJP, Hinton GE. Visualizing High-Dimensional Data Using t-SNE. Journal of Machine Learning Research. 2008;9(nov):2579-2605. Available from: https://jmlr.org/papers/volume9/vandermaaten08a/vandermaaten08a.pdf

3. Papp L, Spielvogel CP, Rausch I, Hacker M, Beyer T. Personalizing Medicine Through Hybrid Imaging and Medical Big Data Analysis. Front Phys [Internet]. 2018 Jun 7;6. Available from: https://www.frontiersin.org/article/10.3389/fphy.2018.00051/full

4. Han J, Pei J, Kamber M. Data Mining: Concepts and Techniques [Internet]. Elsevier Science; 2011. (The Morgan Kaufmann Series in Data Management Systems). Available from: https://books.google.at/books?id=pQws07tdpjoC

5. Papp L, Spielvogel CP, Grubmüller B, Grahovac M, Krajnc D, Ecsedi B, et al. Supervised machine learning enables non-invasive lesion characterization in primary prostate cancer with [68Ga]Ga-PSMA-11 PET/MRI. Eur J Nucl Med Mol Imaging [Internet]. 2020 Dec 19; Available from: http://link.springer.com/10.1007/s00259-020-05140-y

6. Amin A, Anwar S, Adnan A, Nawaz M, Howard N, Qadir J, et al. Comparing Oversampling Techniques to Handle the Class Imbalance Problem: A Customer Churn Prediction Case Study. IEEE Access. 2016;4(October):7940–57.

7. Langarizadeh M, Moghbeli F. Applying Naive Bayesian Networks to Disease Prediction: a Systematic Review. Acta Inform Medica [Internet]. 2016;24(5):364. Available from: http://www.scopemed.org/?mno=247785

8. Papp L, Pötsch N, Grahovac M, Schmidbauer V, Woehrer A, Preusser M, et al. Glioma survival prediction with combined analysis of in vivo 11C-MET PET features, ex vivo features, and patient features by supervised machine learning. J Nucl Med. 2018;59(6):892–9.

9. Gao X, Chu C, Li Y, Lu P, Wang W, Liu W, et al. The method and efficacy of support vector machine classifiers based on texture features and multi-resolution histogram from18F-FDG PET-CT images for the evaluation of mediastinal lymph nodes in patients with lung cancer. Eur J Radiol [Internet]. 2015;84(2):312–7. Available from: http://dx.doi.org/10.1016/j.ejrad.2014.11.006

10. Yun J, Park JE, Lee H, Ham S, Kim N, Kim HS. Radiomic features and multilayer perceptron network classifier: a robust MRI classification strategy for distinguishing glioblastoma from primary central nervous system lymphoma. Sci Rep [Internet]. 2019 Dec 5;9(1):5746. Available from: http://www.nature.com/articles/s41598-019-42276-w

11. van der Laan MJ, Polley EC, Hubbard AE. Super Learner. Stat Appl Genet Mol Biol [Internet]. 2007 Jan 16;6(1). Available from: https://www.degruyter.com/view/j/sagmb.2007.6.issue-1/sagmb.2007.6.1.1309/sagmb.2007.6.1.1309.xml

12. Stehman S V. Selecting and interpreting measures of thematic classification accuracy. Remote Sens Environ [Internet]. 1997 Oct;62(1):77–89. Available from: https://linkinghub.elsevier.com/retrieve/pii/S0034425797000837
